# The NAC transcription factor family in *Eucommia ulmoides*: Genome-wide identification, characterization, and network analysis in relation to the rubber biosynthetic genes

**DOI:** 10.3389/fpls.2023.1030298

**Published:** 2023-04-03

**Authors:** Shuwen Zhang, Tingting Xu, Yongyu Ren, Lianjun Song, Zhao Liu, Xiangyang Kang, Yun Li

**Affiliations:** ^1^ State Key Laboratory of Tree Genetics and Breeding, Beijing Forestry University, Beijing, China; ^2^ National Engineering Research Center of Tree Breeding and Ecological Restoration, College of Biological Sciences and Technology, Beijing Forestry University, Beijing, China; ^3^ Key Laboratory of Genetics and Breeding in Forest Trees and Ornamental Plants, Ministry of Education, Beijing Forestry University, Beijing, China; ^4^ Beijing Laboratory of Urban and Rural Ecological Environment, Beijing Forestry University, Beijing, China; ^5^ Weixian Eucommia National Forest Tree Germplasm Repository, Weixian Forestry Cultivation Base of Superior Species, Hebei, China

**Keywords:** *Eucommia ulmoides*, NAC transcription factor, gene family, gene expression, *Eucommia* rubber (Eu-rubber), hormone response

## Abstract

The *NAC* transcription factor family is a large plant gene family, participating in plant growth and development, secondary metabolite synthesis, biotic and abiotic stresses responses, and hormone signaling. Eucommia ulmoides is a widely planted economic tree species in China that can produce trans-polyisoprene: Eucommia rubber (Eu-rubber). However, genome-wide identification of the NAC gene family has not been reported in *E. ulmoides*. In this study, 71 NAC proteins were identified based on genomic database of *E. ulmoides*. Phylogenetic analysis showed that the EuNAC proteins were distributed in 17 subgroups based on homology with NAC proteins in Arabidopsis, including the E. ulmoides-specific subgroup Eu_NAC. Gene structure analysis suggested that the number of exons varied from 1 to 7, and multitudinous *EuNAC* genes contained two or three exons. Chromosomal location analysis revealed that the *EuNAC* genes were unevenly distributed on 16 chromosomes. Three pairs of genes of tandem duplicates genes and 12 segmental duplications were detected, which indicated that segmental duplications may provide the primary driving force of expansion of *EuNAC*. Prediction of cis-regulatory elements indicated that the *EuNAC* genes were involved in development, light response, stress response and hormone response. For the gene expression analysis, the expression levels of *EuNAC* genes in various tissues were quite different. To explore the effect of *EuNAC* genes on Eu-rubber biosynthesis, a co-expression regulatory network between Eu-rubber biosynthesis genes and *EuNAC* genes was constructed, which indicated that six *EuNAC* genes may play an important role in the regulation of Eu-rubber biosynthesis. In addition, this six *EuNAC* genes expression profiles in E. ulmoides different tissues were consistent with the trend in Eu-rubber content. Quantitative real-time PCR analysis showed that *EuNAC* genes were responsive to different hormone treatment. These results will provide a useful reference for further studies addressing the functional characteristics of the NAC genes and its potential role in Eu-rubber biosynthesis.

## Introduction

NAC [for NAM (no apical meristem), ATAF, and CUC (cup-shaped cotyledon)] proteins are one of the largest plant transcription factors (TF) families. NAC TFs are characterized by a conserved N-terminal NAC domain comprising approximately 150 amino acids and a diversified C-terminal (Ng et al., 2018). The DNA binding NAC domain is divided into five sub-domains designated A–E, which are relevant to DNA binding, dimer formation and multiple other functions (Ooka et al., 2003; Ernst et al., 2004). In addition, compared with subdomains B and E, subdomains A, C, and D are highly conserved (Jensen et al., 2010). The C-terminus has transcriptional activation or transcriptional repression activity (Mohanta et al., 2020). With the development of high-throughput sequencing technology, it has become possible to use genome and transcriptome data to identify and screen all *NAC* family genes in species. The members of the *NAC* gene family has been widely identified and studied in many species, such as *Arabidopsis thaliana* (Ooka et al., 2003), *Oryza sativa* (Nuruzzaman et al., 2010), *Vitis vinifera* (Wang et al., 2013), *Coffea canephora* (Dong et al., 2019), and *Populus trichocarpa* (Hu et al., 2010).

NAC transcription factors play an important role in regulating plant growth and development. For example, secondary wall-associated NAC (*SWN*) transcription factors play critical roles in regulating secondary cell wall formation and development (Zhou et al., 2014; Endo et al., 2015; Zhang et al., 2020; Zhong et al., 2021a). *AtNAC1* and *AtNAC2* are involved in lateral root development by downregulating auxin signals in Arabidopsis (Guo et al., 2005). At the same time, *NAC* genes also play a key role in responses to biotic and abiotic stresses. *AtNAC019*, *AtNAC055*, and *AtNAC072* can respond to various abiotic stresses and hormonal treatments, such as dehydration, cold, salinity, mechanical damage, and jasmonic acid (JA) and abscisic acid (ABA) (Tran et al., 2004). In addition, the involvement of NAC transcription factors in plant secondary metabolite biosynthesis is attracting increasing attention (Wang et al., 2018). *CsNAC7* positively regulates the caffeine synthase gene *yhNMT1* and promotes caffeine accumulation in *Camellia sinensis* (Ma et al., 2022). The overexpression of *SmNAC2* reduces the content of tanshinone in *Salvia miltiorrhiza*, but silencing *SmNAC2* promotes the accumulation of tanshinone (Zhang et al., 2021).

Polyisoprene, an isoprene (C5H8) polymer, is the primary chemical constituent in natural rubber. Based on the chemical structures of two isoprene isomers, natural rubber can be classified as cis-polyisoprene (CPI) and trans-polyisoprene (TPI) (Baboo et al., 2011). Over 2000 different plant species produce CPI, including *Hevea brasiliensis*, *Lactuca sativa*, *Taraxacum kok-saghyz* and *Parthenium argentatum*. (Mooibroek and Cornish, 2000; van Beilen and Poirier, 2007). However, only a few plants can produce trans-rubber, *Eucommia ulmoides* is a widely known tree species that can produce TPI: Eu-rubber (Roth et al., 1985; Schlesinger and Leeper, 2002). Eu-rubber is especially enriched in the leaves, bark, and peels of *E. ulmoides* trees (Nakazawa et al., 2009; Wuyun et al., 2018). Compared with CPI, Eu-rubber has unique features, good insulation and resistance to acids and alkalis; moreover, it also has great potential for application in biomedicine, textiles, aerospace and other fields. (Wang et al., 2020). Therefore, *E. ulmoides* is an ideal material for studying the biosynthesis of TPI. Although the biosynthetic pathway of Eu-rubber has been widely studied, the molecular mechanisms regulating the biosynthesis of Eu-rubber remain unclear. However, the *NAC* gene family of *E. ulmoides* has not been systematically analyzed, and its effect on the biosynthesis of TPI is still unclear.

In this study, we identified 71 *E. ulmoides NAC* genes and divided them into 17 subgroups, including an *E. ulmoides*-specific subgroup *Eu_NAC*. A comprehensive analysis of gene structure, motif composition, chromosomal distribution, gene duplication, phylogenetic, and cis-acting elements in promoters and syntenic relationships was completed. In addition, the expression of *EuNAC* genes in different tissues was also analyzed. The co-expression network of *EuNAC* genes and Eu-rubber biosynthesis genes and the response to various hormones were also analyzed. The results of this study will provide a platform to identify the biological function of *NAC* genes in *E. ulmoides* in the future and will be helpful for further study of the functional characteristics of *EuNAC* genes in the mechanism of Eu-rubber biosynthesis.

## Materials and methods

### Identification of *EuNAC* genes in the *E. ulmoides*

The protein sequence was obtained from the *E. ulmoides* genome. The genome accession number is PRJNA599775 in NCBI database (https://www.ncbi.nlm.nih.gov/). First, we downloaded the HMM profile of the NAM domain (PF02365) from the Pfam database (http://pfam.sanger.ac.uk/). Then, the HMMER 3.2.1 program was used to identify the *NAC* protein in *the E. ulmoides* genome (Li et al., 2020). The default setting was used, and the cut-off was 0.01. Finally, the NAM domain of all candidate *NAC* genes was determined through CDD (http://www.ncbi.nlm.nih.gov/cdd) and SMART databases (http://smart.emblheidelberg.de/). Seventy-one putative *NAC* genes were identified. Meanwhile, the physical and chemical parameters of the *EuNAC* proteins were predicted by ProtParam (http://web.expasy.org/protparam/), including the CDS (coding sequence) length, protein length, molecular weights (MW), isoelectric points (PI) aliphatic index, and grand average of hydropathicity (GRAVY). Subcellular localization of all *EuNAC* proteins was performed using Euk-mPLoc 2.0 (http://www.csbio.sjtu.edu.cn/bioinf/euk-multi-2/).

### Phylogenetic analysis and multiple alignments

The NAC protein sequences of Arabidopsis were downloaded from the Arabidopsis genome TAIR 11 (https://www.arabidopsis.org/). Multiple sequence alignments of *E. ulmoides* and Arabidopsis NAC proteins were performed using MUSCLE with default parameters. The phylogenetic and molecular evolutionary analyses were conducted using MEGA (version X) (Sudhir et al., 2018). The neighbor-joining (NJ) method was selected for constructing the phylogenetic tree with 1000 bootstrap replications. The phylogenetic tree was visualized in the EvolView program (https://www.evolgenius.info//evolview/). All the identified *EuNAC* genes were assigned into different groups based on the classification of the Arabidopsis *NACs*.

### Gene structure and motif analysis

Gene structure was investigated using the TBtools software (version 1.098696). The MEME online program (http://meme.nbcr.net/meme/intro.html) for protein sequence analysis was used to identify conserved motifs in the identified *EuNACs* proteins, with the maximum number of motifs set to 10 and the width of each motif ranging from 6 to 50. The TBtools software was used to integrate phylogenetic trees, conserved motifs, and gene structure results (Chen et al., 2020).

### Chromosomal location, gene duplication, and synteny analysis with several plant species

The chromosomal position of *NAC* genes was obtained from the *E. ulmoides* genome annotations using TBtools. The chromosomal map of *E. ulmoides NAC* genes was visualized by MG2C.10 (http://mg2c.iask.in/mg2c_v2.1/). *EuNAC* gene duplication events was examined by using MCScanX software with default parameters (Wang et al., 2012). Dual Synteny Plotter software (https://github.com/CJ-Chen/TBtools/) was used to analyze the homology of the NAC gene between *E. ulmoides* and *A.thaliana*, *C.canephora*, *V.vinifera*, *H.brasiliensis*, *O.sativa*, and *S.bicolor*, respectively. TBtools software was used to visualize the obtained results, the obtained *NAC* homologous pairs are highlighted (Chen et al., 2020). The parameters non-synonymous mutations (Ka), synonymous mutations (Ks) and estimated evolutionary constraints (Ka/Ks) among the *EuNACs* genes were calculated using TBtools.

### Analysis of cis-acting elements in *EuNACs* promoters

The promoter refers to the region upstream sequence of the transcription start site (Shafee and Lowe, 2017). At present, we are unable to identify the transcription start site of genes in *E.ulmoides*, so we used the upstream 2000 bp sequence of the translational start codon (ATG) of *EuNAC* genes and Eu-rubber biosynthesis pathway genes, and the sequences were submitted to PlantCARE database (http://bioinformatics.psb.ugent.be/webtools/plantcare/html/) to predict cis-acting elements. This means that we may have explored 5’ UTR or the sequences analyzed were only part of the promoter regulatory region.

### Expression patterns of *EuNACs* in different tissue based on the public RNA-seq data sets

To survey the expression patterns of *EuNAC* genes in different tissues, the transcriptome data of *E. ulmoides* in various tissues (Leaf, Xylem, Seed, and Peel) were obtained from the NCBI sequence read archive (SRX7525252-54, SRX7532003-05, SRX7531725-27, and SRX7533248-50). The transcript abundance of *E. ulmoides* genes was calculated as fragments per kilobase of exon model per million mapped reads (FPKM). The EuNAC genes expression level was presented based on the transformed data of log_2_ (FPKM+1) values. A clustered heat map was drawn using Tbtools software, the approach of clustering analysis is hierarchical clustering. (Chen et al., 2020).

### Gene Co-Expression Network Construction

To study the relationship between *EuNAC* genes and Eu-rubber biosynthesis pathway genes in *E. ulmoides*, we used the FPKM of these genes to construct the network. Pearson’s correlation analysis was performed using the OmicStudio tools at https://www.omicstudio.cn/tool/62 (Lyu et al., 2023). Genes with a Pearson’s correlation coefficient within the appropriate range (r ≥0.6 or ≤−0.6) and p < 0.05 were selected to generate a co-expression network using Cytoscape (version 3.9.0). A positive value represented a positive correlation, and a negative value represented a negative correlation. The connectivity degree of genes was calculated using the Cytoscape software. The node size was positively correlated with the degree of the connectivity of genes. Nodes were colored red for positive regulation and blue for negative regulation.

### Plant materials and hormone treatments

The *E. ulmoides* seeds were collected from 20-year-old diploid *E. ulmoides* trees at Beijing Forestry University, Beijing, China. The *E. ulmoides* were cultured in a growth chamber with a 16-h light/8-h dark cycle at 28°C. Studies reported that applications of exogenous hormones, such as 2-(3,4-dichlorophenoxy)-triethylamine (DCPTA), gibberellin (GA_3_) and brassinolide (BR) increased Eu-rubber concentration in *E. ulmoides* leaves and spaying BR at 5 mg/L, DCPTA at 500 mg/L and GA_3_ at 300 mg/L was the optimal treatment concentration (Liu H. et al., 2018). To examine the effect of hormones on the expression of *EuNAC* genes, 5-month-old seedling leaves of *E. ulmoides* were sprayed with 300 mg/L GA_3_, 5 mg/L 1% BR, and 500 mg/L DCPTA until there is liquid dripping. Spray water was used as the control treatment. The leaves were sampled at 0, 3, 6, 12, and 24 h after hormone treatments, frozen in liquid nitrogen, and finally stored at −80°C for RNA extraction. There were three independent replicates for each treatment.

### RNA extraction, cDNA synthesis, and qRT-PCR gene expression analysis

The RN38-EASYspin Plus Kit (Aidlab Biotechnologies Co., Ltd) was used to extract total RNA according to the manufacturer’s instructions. A NanoDrop ND-2000 (Thermo Scientific, USA) spectrophotometer and 1% agarose gel electrophoresis were used to detect the RNA quality of all samples. The PC54-TRUEscript RT kit (Aidlab Biotechnologies Co., Ltd) was used to synthesize first-strand cDNA. The real-time polymerase chain reaction (RT-PCR) was accomplished using SYBR^®^ Green Premix Pro Taq HS qPCR Kit (Rox Plus) AG11718 (Accurate Biotechnology (Hunan) Co., Ltd.) using an Applied Biosystems 7500 Fast instrument (AB Ltd., USA). The qRT-PCR master mix included 10 µL 2× SYBR Green Pro Taq HS Premix (ROX Plus), 0.4 µL forward primer, 0.4 µL reverse primer, 2 µL cDNA template, and 7.2 µL RNase-free ddH_2_O. The RT-PCR was performed using 40 cycles under the following conditions: 95°C for 1 min for pre-degeneration, 95°C for 5 s for degeneration, and 60°C for 30 s for the extension. Afterward, the samples were heated to 95°C for 15 s and then 60°C for 1 min for dissolution curve analysis. Three technical replicates and three biological replicates were used for each sample and randomly selected genes. The primers used in the present study were designed using primer3plus (http://www.primer3plus.com/) and are listed in **Supplementary Table S9**. *UBCE2* was chosen as the reference gene (Ye et al., 2018), and the −2^–△△Ct^ method was used to calculate the relative gene expression levels (Schmittgen and Livak, 2008).

### Statistical analysis

The statistical analyses were conducted using SPSS Statistics version 20 software, and the Student t-test was selected for significant difference analysis (* p < 0.05 and ** p < 0.01).

## Results

### Identification of the *EuNAC* genes in *E. ulmoides*

Based on the genomic information of *E. ulmoides*, 74 putative *NAC* genes were obtained using HMM. According to the results of CDD and SMART databases, three genes without NAC and NAM domains were eliminated, leaving 71 *EuNAC* genes, which were named *EuNAC1* to *EuNAC71* according to their chromosomal position (**Supplementary Table S1**).

Gene characteristics, including the length of the CDS, the length of the protein sequence, the protein MW, pI, GRAVY, aliphatic index, and the subcellular localization, were examined (**Supplementary Table S2**). The protein sequence length of all EuNAC proteins ranged from 86 (*EuNAC49*) to 617 (*EuNAC65*) amino acids. The MW of the proteins was between 9821.26 (*EuNAC49*) and 70458.62 (*EuNAC65*) Da, and the pI ranged from 4.51 (*EuNAC20*) to 10.01 (*EuNAC5*).

The aliphatic index varied from 47.7 (*EuNAC35*) to 100.0 (*EuNAC13*), which suggested that these predicted EuNAC proteins contained rich aliphatic amino acids. The GRAVY values of the EuNAC proteins were negative, except for *EuNAC13*, which has a positive value, indicating that most EuNAC proteins were hydrophobic. The predicted subcellular localization results showed that 69 EuNAC proteins were located in the nuclear region, whereas *EuNAC13* was located in the nuclear or mitochondrion, and *EuNAC*60 was located in the nuclear or cytoplasm.

### Phylogenetic analysis and classification of *EuNAC* genes

**Figure 1:**
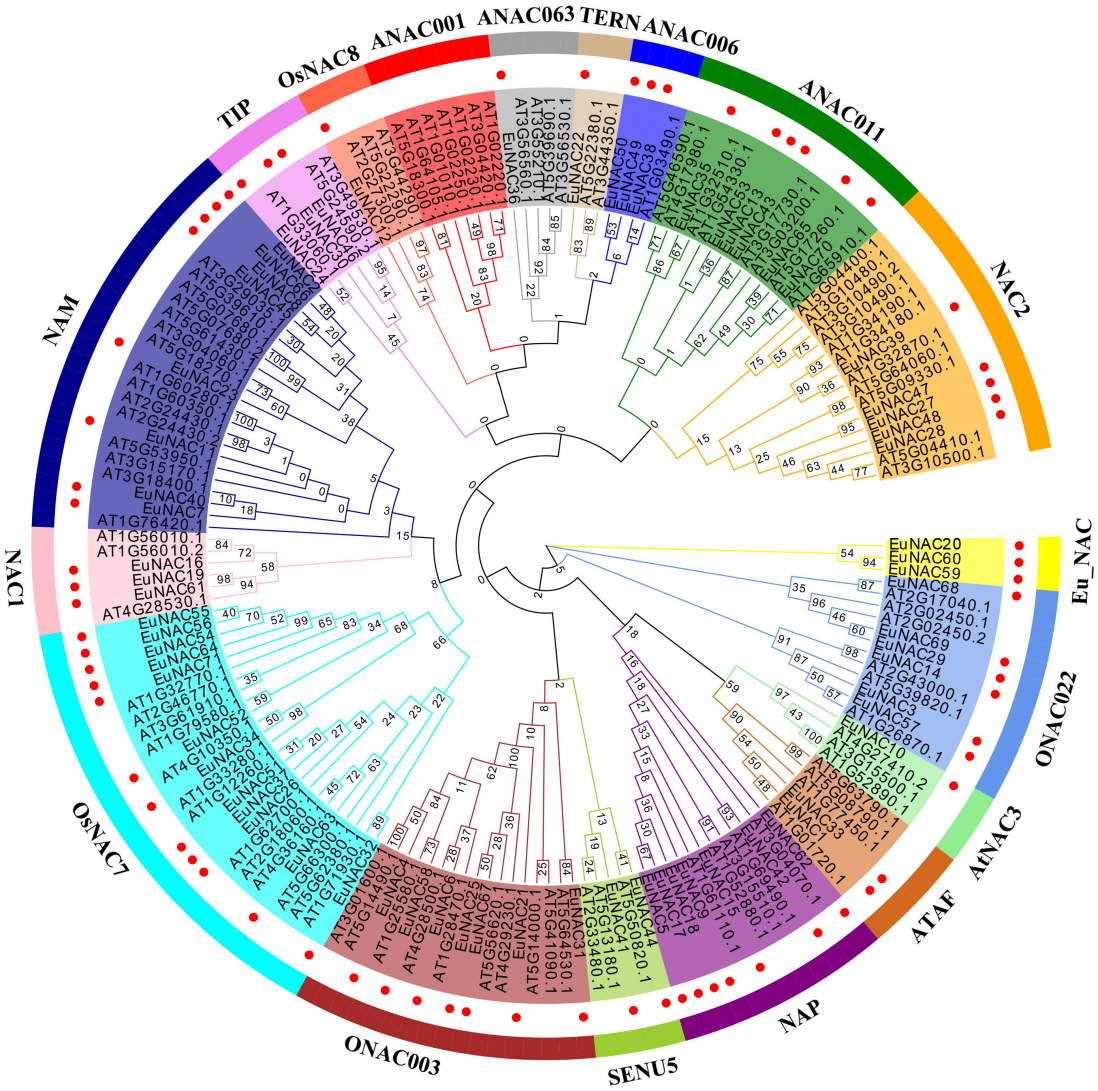
Phylogeny of the NAC TFs of *Arabidopsis thaliana* and *Eucommia ulmoides*. The tree branched the NAC proteins into different groups illustrated by different colored clusters within the clade. Markers of genes with red circles were *EuNAC.* The phylogenetic tree was prepared using the neighbor-joining (NJ) method with 1000 bootstrap replicates using MEGA 6.0.

To explore the evolutionary relationship of the *NAC* gene family in *E. ulmoides*, an unrooted neighbor-joining (NJ) tree (with 1000 bootstraps) was constructed using the amino acid sequence alignment of NAC proteins from *E. ulmoides* and *A. thaliana* (**Figure 1**). The 71 *E. ulmoides NAC* genes could be divided into 17 subgroups: the *ONAC022*, *AtNAC3*, *ATAF*, *NAP*, *SENU5*, *ONAC003*, *TIP*, *OsNAC8*, *ANAC063*, *TERN*, *ANAC006*, *NAC2*, *ANAC011*, *NAC1*, *NAM*, *OsNAC7* subgroups and an *E. ulmoides*-specific subgroup, named *Eu_NAC*. However, in *E. ulmoides*, no *NAC* members were detected from the *ANAC001* subgroup. The subgroups *AtNAC3*, *OsNAC8*, *TERN*, and *ANAC063* each contained only one EuNAC protein, and only two EuNAC proteins belonged to the subgroups *NAP* and *SENU5* each, whereas the subgroup *OsNAC7* contained the greatest number of EuNAC proteins (12).

### Gene structures, domain, and putative motifs characterization of *EuNAC* genes

**Figure 2:**
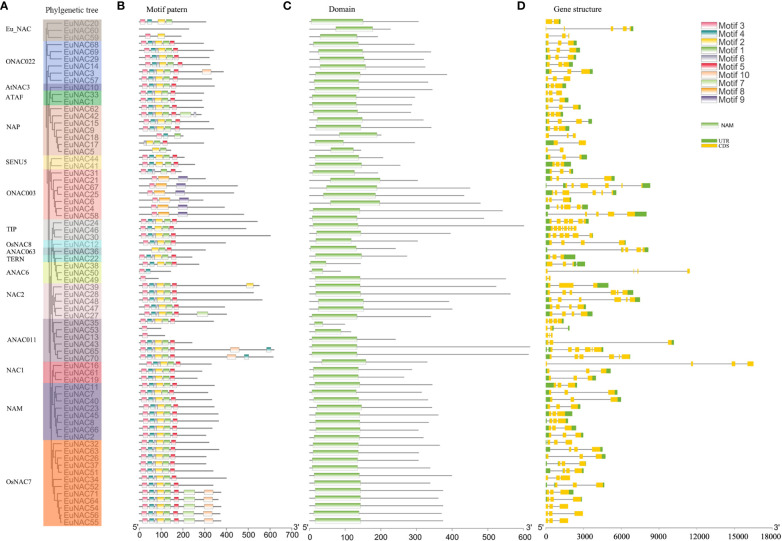
Phylogenetic relationships, domin, gene structure and architecture of conserved protein motifs in NAC genes from *E. ulmoides*. **(A)** Estimated phylogeny of *EuNAC* genes. **(B)** Different motifs were represented by diferent colors. The black lines represented the non-conserved sequences. Lengths of motifs for each *EuNAC* protein were displayed proportionally. **(C)** NAC domains were represented by green. **(D)** UTR were represented by green, CDS were represented by yellow. Nucleic acid lengths are indicated by the scale at the bottom.

**Figure 3:**
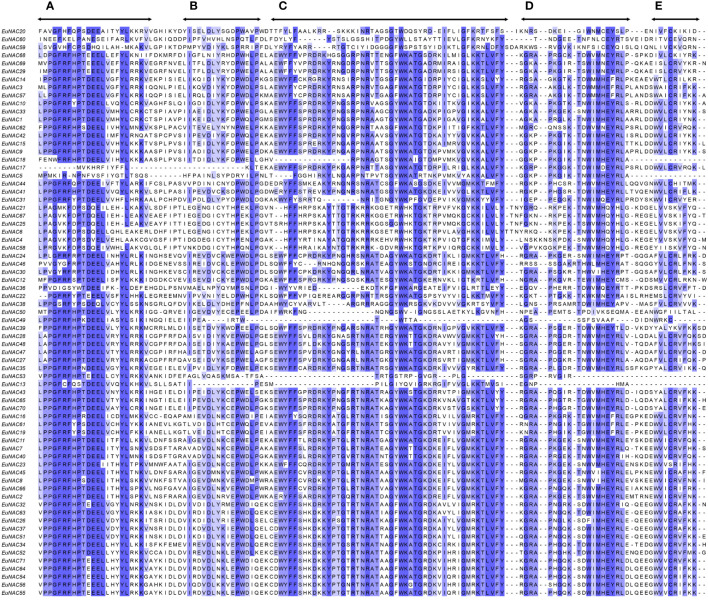
Sequence alignment of two groups of EuNAC domains amino acid sequences. Subdomains **(A–E)** are shown by arrows above the sequences. Overall conserved amino acids were shaded in blue.

The NAC proteins comprised an N-terminal NAC domain and a long transcriptional activation region at the C-terminal (**Figure 2C**; **Supplementary Table S3**). As shown in **Figure 3**, All the *EuNAC* proteins contained five NAC subdomains (A–E), except *EuNAC13*, *EuNAC43*, *EuNAC5*, and *EuNAC18*, which lacked domains E.

To reveal the protein structural diversification of EuNAC proteins, 10 conserved motifs were identified by MEME (**Figure 2B**). The amino acid sequences of each motif are listed in **Figure S1**. The lengths of these conserved motifs ranged from 11 to 57 amino acids and were highly diverse. Motifs 1–6 were the most conserved motifs in EuNAC proteins, Motif 8 and Motif 9 only appeared in *ONAC003*. Among 71 EuNAC proteins, *EuNAC49*, *EuNAC53*, and *EuNAC13* only had one type of motif, whereas *EuNAC71*, *EuNAC64*, *EuNAC54*, *EuNAC56*, *EuNAC55*, *EuNAC65*, *EuNAC70*, and *EuNAC42* contain the largest number of motifs (eight types). The motifs of *EuNAC* members within the same subgroups show similar patterns, indicating that NAC proteins placed in the same group probably have similar functions. However, these motifs’ specific biological functions are unclear and need further study.

To obtain more insights into the evolution of the NAC family in *E. ulmoides*, the structural features of all the identified *EuNAC* genes were analyzed. As shown in **Figure 2D**, among the *EuNAC* genes, all of these contain exons; only *EuNAC5*, *EuNAC17*, *EuNAC20*, and *EuNAC49* had one intron and two exons, and over half (40, 56.34%) had two introns and three exons, and only *EuNAC46* genes had seven introns and eight exons (**Figure 2D**; **Supplementary Table S4**). Most genes from the same subgroup had a similar exon/intron structure; for example, 87.50% of *NAM* subgroup genes in *E. ulmoides* had three exons, and all of the *ONAC022* subgroups genes had three exons.

### Chromosome distribution and synteny analysis of *EuNAC* genes

**Figure 4:**
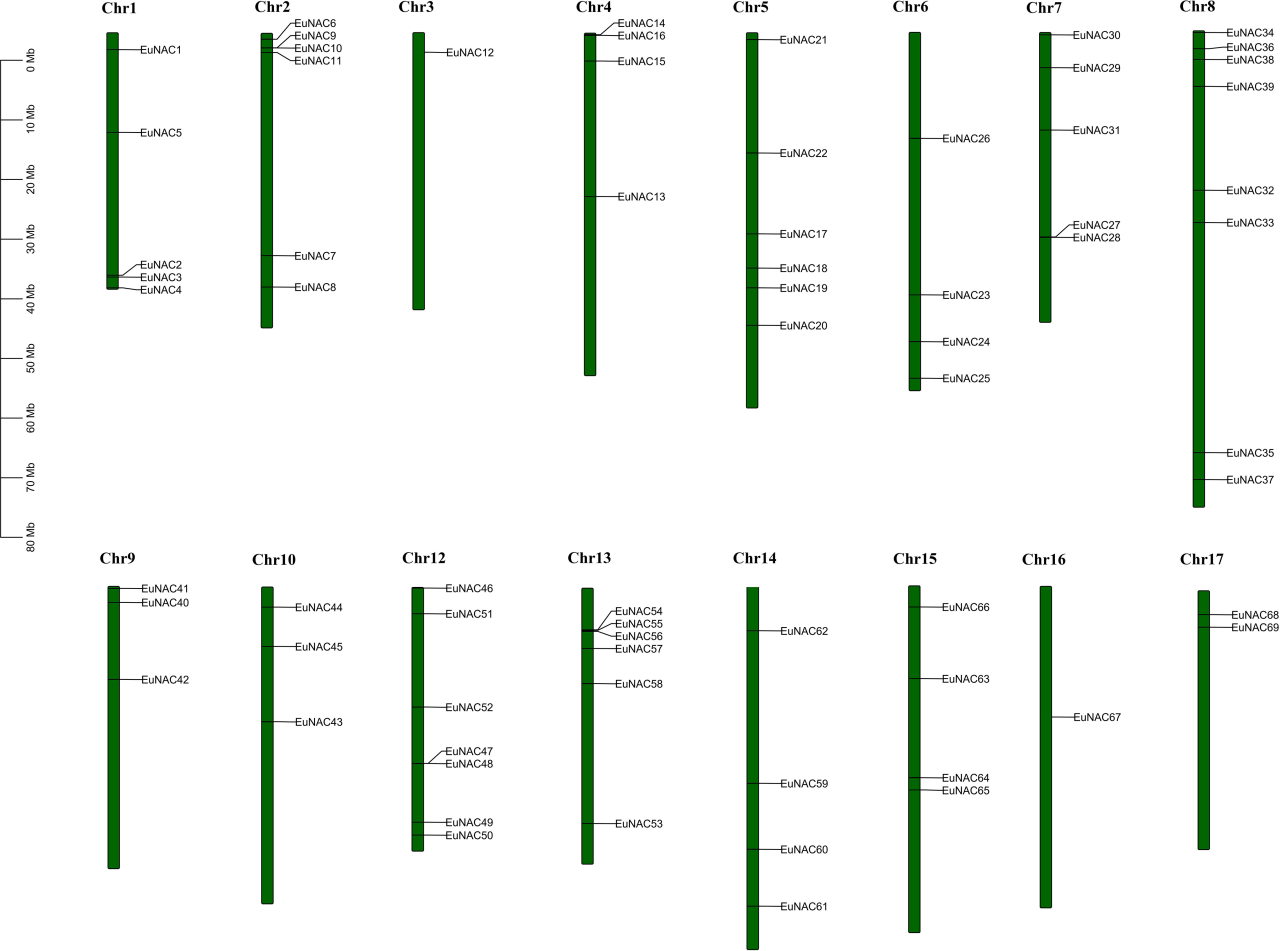
Gene Location on chromosome of *E. ulmoides*. Vertical bars represent the chromosomes of *E. ulmoides*. The chromosome number is to the up of each chromosome. The scale on the left represents chromosome length.

A total of 71 *EuNAC* genes were unevenly distributed on 16 chromosomes of *E. ulmoides*, there was no *EuNAC* located on chromosome 11. Chromosome 8 had the largest number of *EuNAC* genes (8, 25.35%), and chromosome 3 only harbored *EuNAC12* (**Figure 4**). The *E. ulmoides*-specific *NAC* genes are distributed on chromosomes 5 and 14. The *EuNAC70* and *EuNAC71* genes mapped to the scaffolds of the *E. ulmoides* genome.

**Figure 5:**
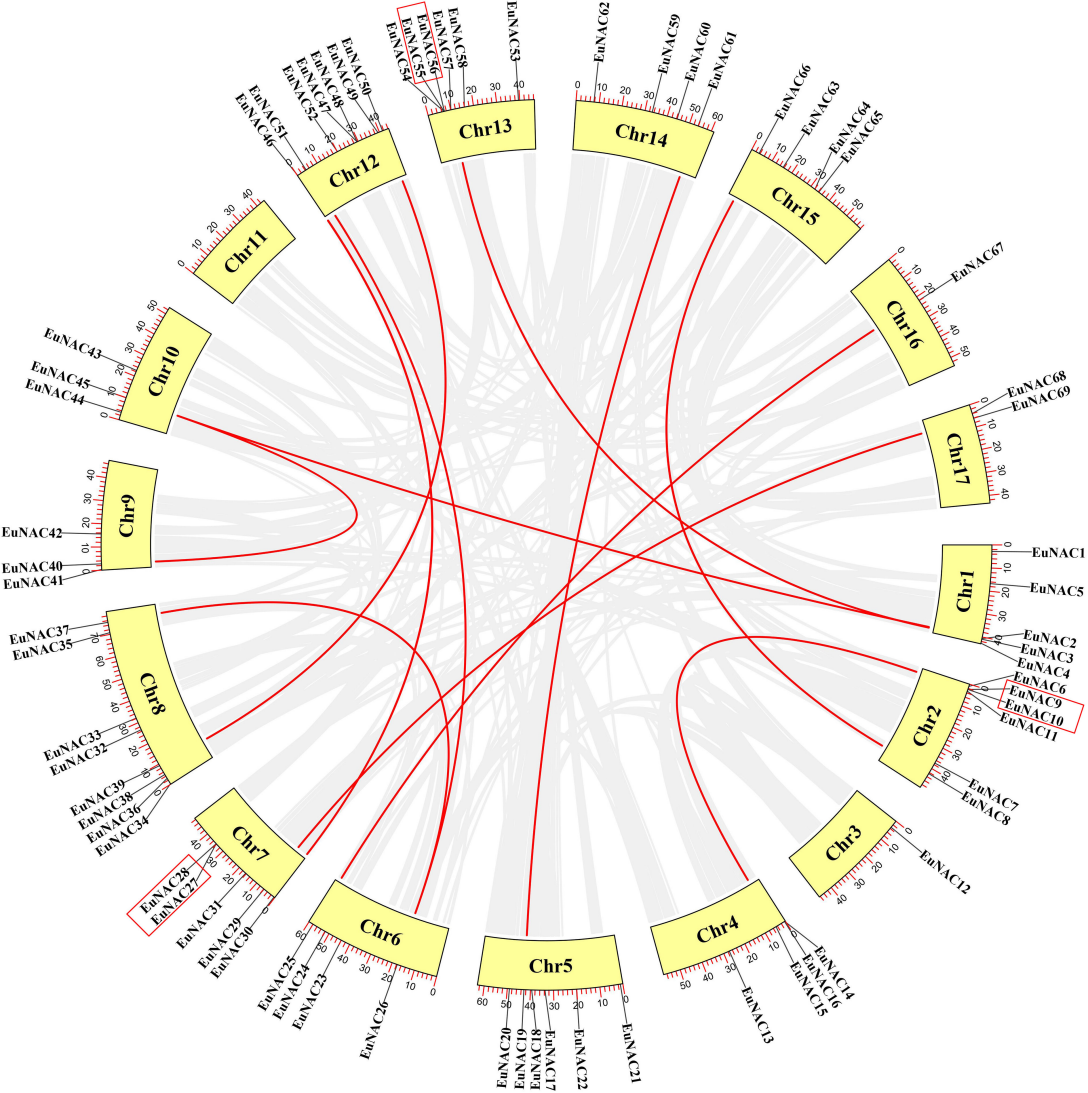
Schematic representations for the chromosomal distribution and interchromosomal relationships of *EuNAC* genes. Gray lines indicate all synteny blocks in the pineapple genome, and the red lines indicate duplicated NAC gene pairs. The tandem duplicated genes were highlighted with a red rectangle. The red scale bar marked on the chromosome represents the length of the chromosome (Mb). The chromosome number is indicated at the bottom of each chromosome.

Tandem duplication is an essential source for the origin and evolution of multigene families. In this study, only three pairs of genes of tandem duplicates genes in the *EuNAC* gene family were identified: *EuNAC9/10*, *EuNAC27/28*, and *EuNAC 55/56*. These are highlighted with a red rectangle (**Figure 5**). The tandem duplicated genes are presented on chromosomes 2, 7, and 13. There were 12 segmental duplication gene pairs identified in the *E. ulmoides NAC* gene family. In addition, the Ka/Ks values for the *EuNAC* genes in tandem and segmental duplications were calculated to determine the selection type that promoted the evolution of the *EuNAC* family. The Ka/Ks values of segmental duplication gene pairs ranged from 0.11 to 0.33, and those of the three tandem duplication gene pairs varied from 0.15 to 0.55, showing that all the gene pairs have a Ka/Ks ratio <1 (**Supplementary Table S5**). These results indicated that the evolution of *EuNAC* genes is mainly affected under purification selection pressure.

**Figure 6:**
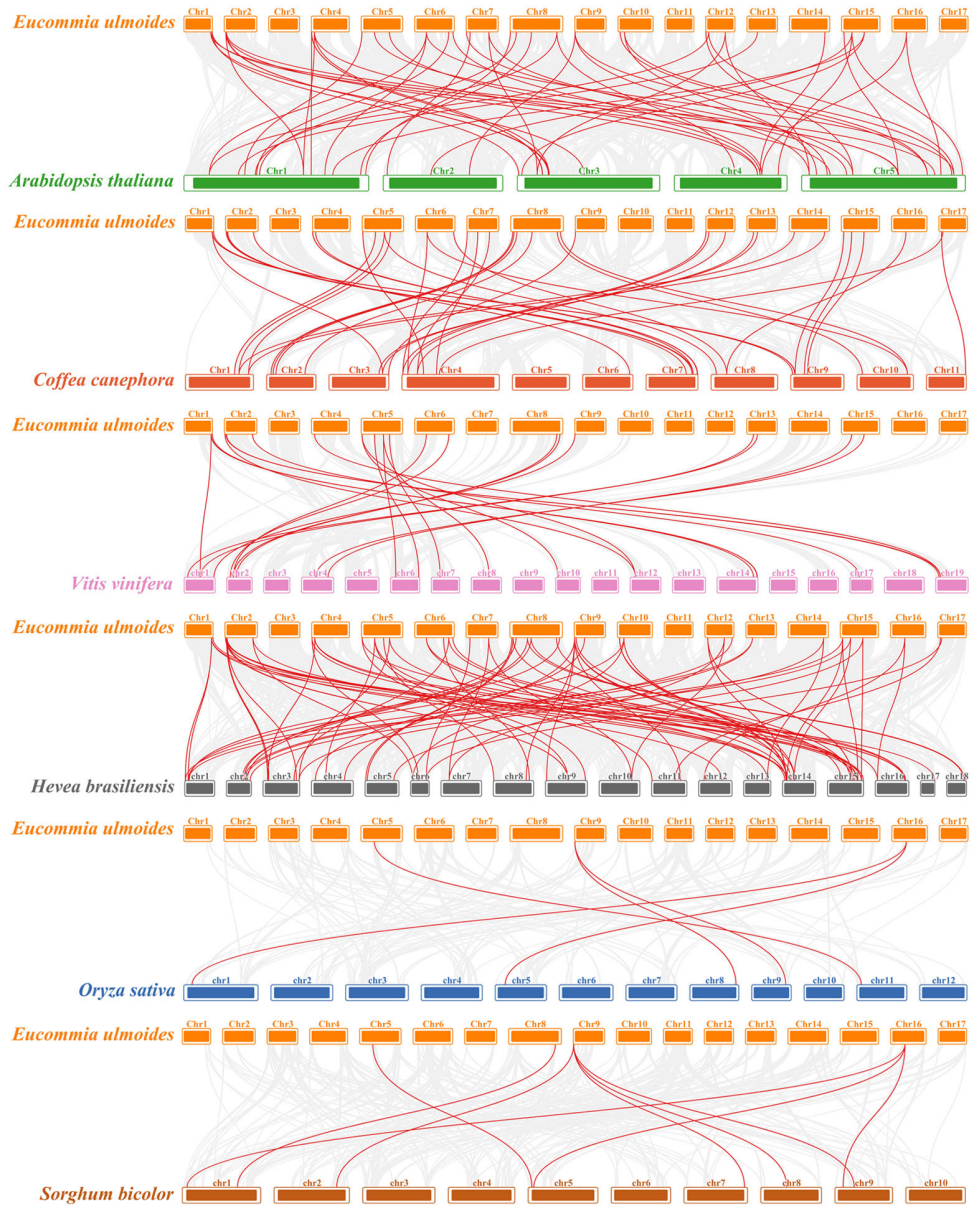
Synteny analysis of NAC genes between *E. ulmoides* and six representative plant species. Gray lines in the background indicate the collinear blocks within the *E. ulmoides* and other plant genomes, whereas the red lines highlight the syntenic NAC gene pairs.

To further explore the evolutionary relationship of the NAC gene family in *E. ulmoides*, we constructed syntenic maps of the *E. ulmoides* compared with six different species including four dicotyledons (*Arabidopsis thaliana*, *Coffea canephora*, *Vitis vinifera* and *Hevea brasiliensis*) and two monocotyledons (*Oryza sativa* and *Sorghum bicolor*). A total of 58, 46, 26, 87, 5, and 10 similar *NAC* gene pairs were identified between *E. ulmoides* and *A.thaliana*, *C.canephora*, *V.vinifera*, *H.brasiliensis*, *O.sativa*, and *S.bicolor*, respectively (**Figure 6**, **Supplementary Table S6**).

### Cis-acting elements in the promoters of *EuNAC* genes

**Figure 7:**
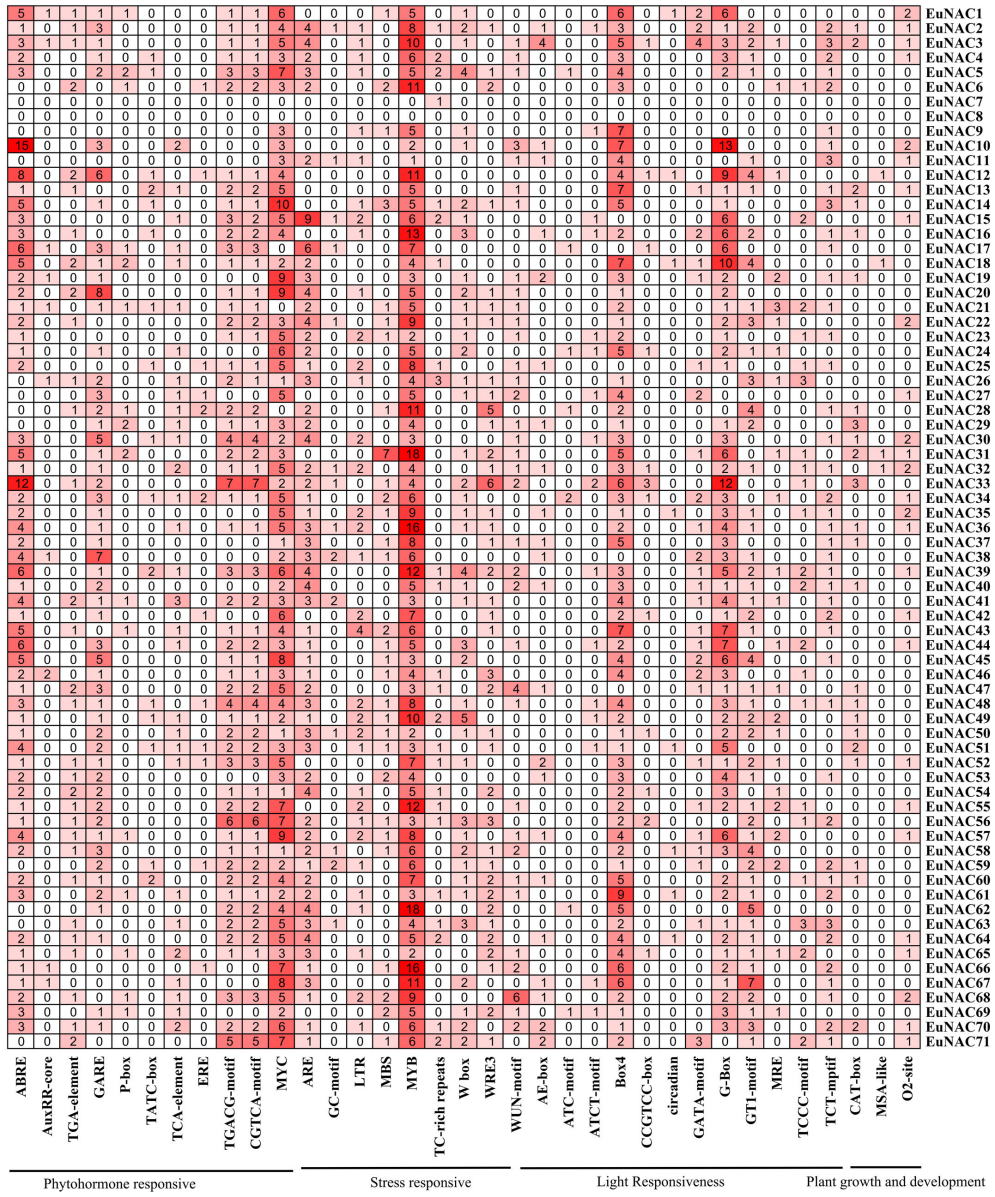
Cis-acting elements in promoter region of *EuNAC* genes in *E. ulmoides*. The number and the shade of red indicate the number of cis-acting element.

To investigate the cis-acting elements of the 71 *EuNAC* genes, a 2000-bp sequence upstream from the translational start codon was analyzed. The cis-acting elements of the *EuNAC* genes contained 35 categories, which were related to phytohormone responsive, stress-responsive, light responsiveness, and plant growth and development (**Figure 7**; **Supplementary Table S7**).

We found that 757 (28.33%) elements, were involved in light responsiveness, including AE-box, ATC-motif, ATCT-motif, Box 4, CCGTCC-box, circadian, GATA-motif, G-box, GT1-motif, and MRE elements. In addition, 70 (2.62%) elements, including CAT-box, MSA-like, and O2-site, were related to plant growth and development. The promoter regions of many *EuNAC* genes contain multiple binding sites involved in stress response. A total of 925 (34.62%) elements were involved in stress response, such as ARE, GC-motif, LTR, MBS, MYB, TC-rich repeats, W box, and WUN-motif. The MYB element was related to drought inducibility, 69 *EuNAC* genes contained the MYB element, which accounted for 16.95% of the total number of cis-acting elements in the *EuNAC* family. Moreover, more than half of the *EuNAC* genes contained the W box element. A total of 2672 elements were predicted in the promoter regions of *EuNAC* genes. Among them, 920 (34.43%) elements were involved in response to plant hormones, such as ABA (ABRE), auxin (AuxRR-core, TGA-element), ethylene (ERE), GA_3_ (P-box, TATC-box, GARE-motif), salicylic acid (TCA-element), and methyl jasmonate (CGTCA-motif, TGACG-motif, MYC). Except for *EuNAC7* and *EuNAC8*, all *EuNAC* genes contained MeJA-responsive elements, which accounted for 18.56% of the total number of cis-acting elements in the *EuNAC* genes. In addition, 58 *EuNAC* genes contained ABA-responsive elements, accounting for 6.62% of the total number of cis-acting elements in the *EuNAC* family, followed by 108 (4.04%) ethylene-responsive elements.

### Expression profiling of *EuNAC* genes in various tissues

**Figure 8:**
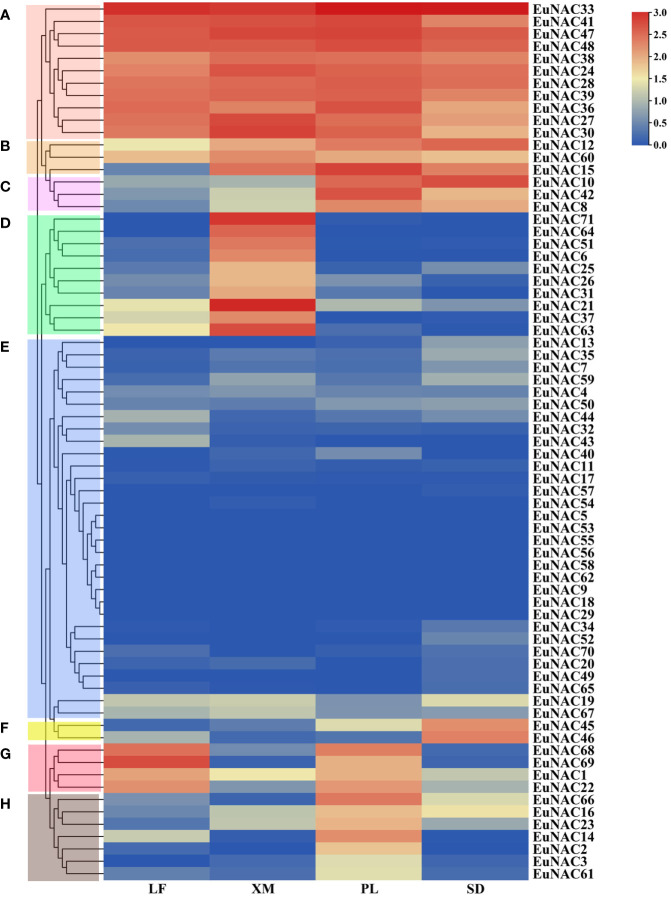
Expression levels of 71 *EuNAC* genes in different tissues. The expression level was presented based on the transformed data of log2 (FPKM+1) values. A total of 71 EuNAC genes were divided into **(A–H)** groups based on their expression levels. LF leaf; PL peel; XM xylem; SD seed.

To determine the expression patterns of individual *NAC* genes in various tissues, a hierarchical clustering heat map was constructed using the public RNA-seq data obtained from NCBI (**Figure 8**). A total of 71 *EuNAC* genes were divided into eight groups based on their expression profiles. The expression levels of *EuNAC* genes were significantly different in diverse tissues. For instance, the FPKM of 28 *EuNAC* genes in Group E was less than 0.1 in all examined organizations, wherein *EuNAC5*, *EuNAC9*, *EuNAC18*, *EuNAC29*, *EuNAC53*, *EuNAC55*, *EuNAC56*, *EuNAC58*, and *EuNAC62* had a FPKM value of 0, reflecting their lack of expression during the sampled stages. In contrast, 11 *EuNAC* genes in Group A were expressed at high levels in all examined tissues. In addition to low expression in leaves, *EuNAC12*, *EuNAC15*, and *EuNAC60* were the highest transcript abundances in the peel, seed, and xylem. Some genes exhibited significant trends in different tissues. Ten genes in the xylem in group D, two genes in the seed in group F (*EuNAC45* and *EuNAC46*), and seven genes in the peel in group H presented high transcript abundances and might play a critical role in the development of distinct tissues. The Group C genes were significantly induced in the peel and seed but rarely expressed in the leaf and xylem, meaning that *EuNAC10*, *EuNAC42*, and *EuNAC8* were associated with fruit development. The transcriptional levels of *EuNAC69*, *EuNAC68*, *EuNAC1*, and *EuNAC22* in Group G were highly expressed in leaves and peel but rarely expressed in the xylem and seeds.

### Co−expression networks between *EuNAC* genes and Eu-rubber biosynthesis genes

It has been reported that at least 52 genes in *E. ulmoides* are involved in the biosynthetic pathway of Eu-rubber (Li et al., 2020). The molecular structure of Eu-rubber is trans-1,4-polyisoprene (TPI), which is synthesized from the precursor ispentenyl diphosphate (IPP) *via* the MVA and MEP pathways.

**Figure 9:**
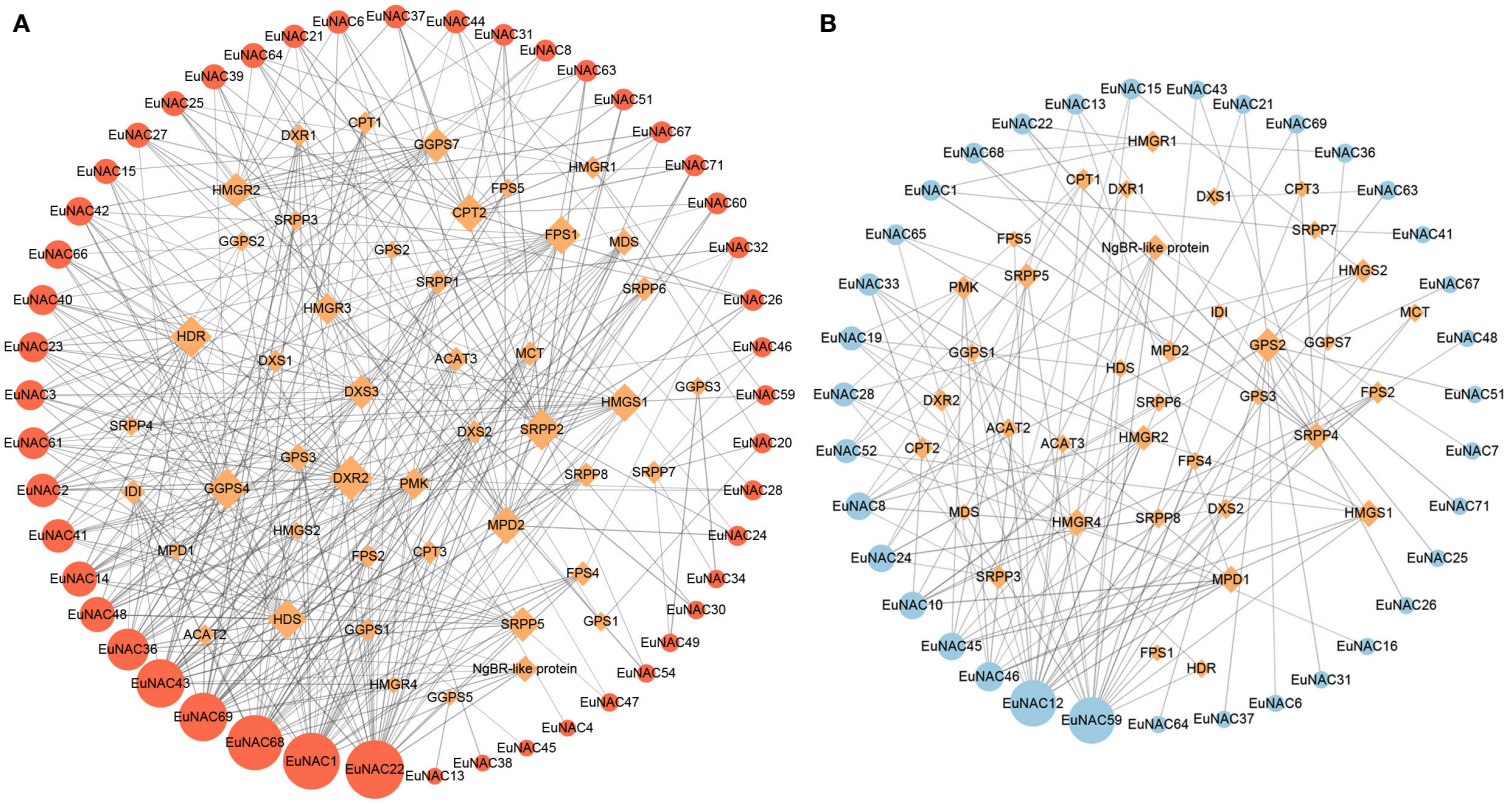
Co-expression networks between *EuNAC* genes and Eu-rubber biosynthesis genes. **(A)** Positive regulatory co-expression network between *EuNAC* genes and Eu-rubber biosynthesis genes. **(B)** Negative regulatory co-expression network between *EuNAC* genes and Eu-rubber biosynthesis genes. Orange diamond nodes represent Eu-rubber biosynthetic structural genes. Red circular nodes represent positively regulated *EuNAC* genes, and blue circular nodes represent negatively regulated *EuNAC* genes. The node size is positively correlated with the degree of the connectivity of the genes. The width of the connecting line is positively related to the correlation between genes.

To understand the possible relationship between *NAC* transcription factors in *E. ulmoides* and Eu-rubber biosynthesis pathway genes, we constructed a co-expression network containing Eu-rubber biosynthesis genes and 71 *EuNAC* genes (**Figure 9**). The screening thresholds were |r| ≥0.60 and p < 0.05. The larger nodes have stronger connectivity degrees, indicating that the genes may be more important. In the positive regulatory co-expression network, we identified 345 pairs correlated between 46 Eu-rubber structural genes and 48 *EuNAC* genes. The degree means the number of Eu-rubber genes associated with *EuNAC*. Statistical analysis showed that 11 *EuNAC* genes had a degree of connection greater than 10. Among them, *EuNAC22* had the highest degree of connection, followed by *EuNAC1*, *EuNAC68*, and *EuNAC69*, all of which have a degree of connection greater than 20, suggesting that these four genes may play a crucial positive role in regulating Eu-rubber biosynthesis. In the negative regulatory co-expression network, we identified 132 pairs correlated between 37 Eu-rubber structural genes and 35 *EuNAC* genes, which was significantly less than the number of positive regulatory genes. Only *EuNAC12* and *EuNAC59* had a connection greater than 10, which might negatively regulate Eu-rubber biosynthesis. The results showed that *EuNAC22*, *EuNAC1*, *EuNAC68*, *EuNAC69*, *EuNAC12*, and *EuNAC59* have the highest degree of connectivity in the co-expression network, indicating that these six genes are probably important in the regulation of Eu-rubber.

### *EuNAC* genes expression in response to hormone treatment

Co-expression analysis was consistent with the results of expression profiles in different tissues, indicating that *EuNAC22*, *EuNAC1*, *EuNAC68*, *EuNAC69*, *EuNAC12*, and *EuNAC59* may play an important role in the regulation of Eu-rubber biosynthesis. To further explore the potential role of these six genes in Eu-rubber biosynthesis, we selected these six genes as candidate genes and paid attention to the expression levels of these genes after exogenous hormone treatment, the expression levels of the *EuNAC* gene family were tested in *E. ulmoides* by qRT-PCR under GA_3_, BR, and DCPTA treatments.

**Figure 10:**
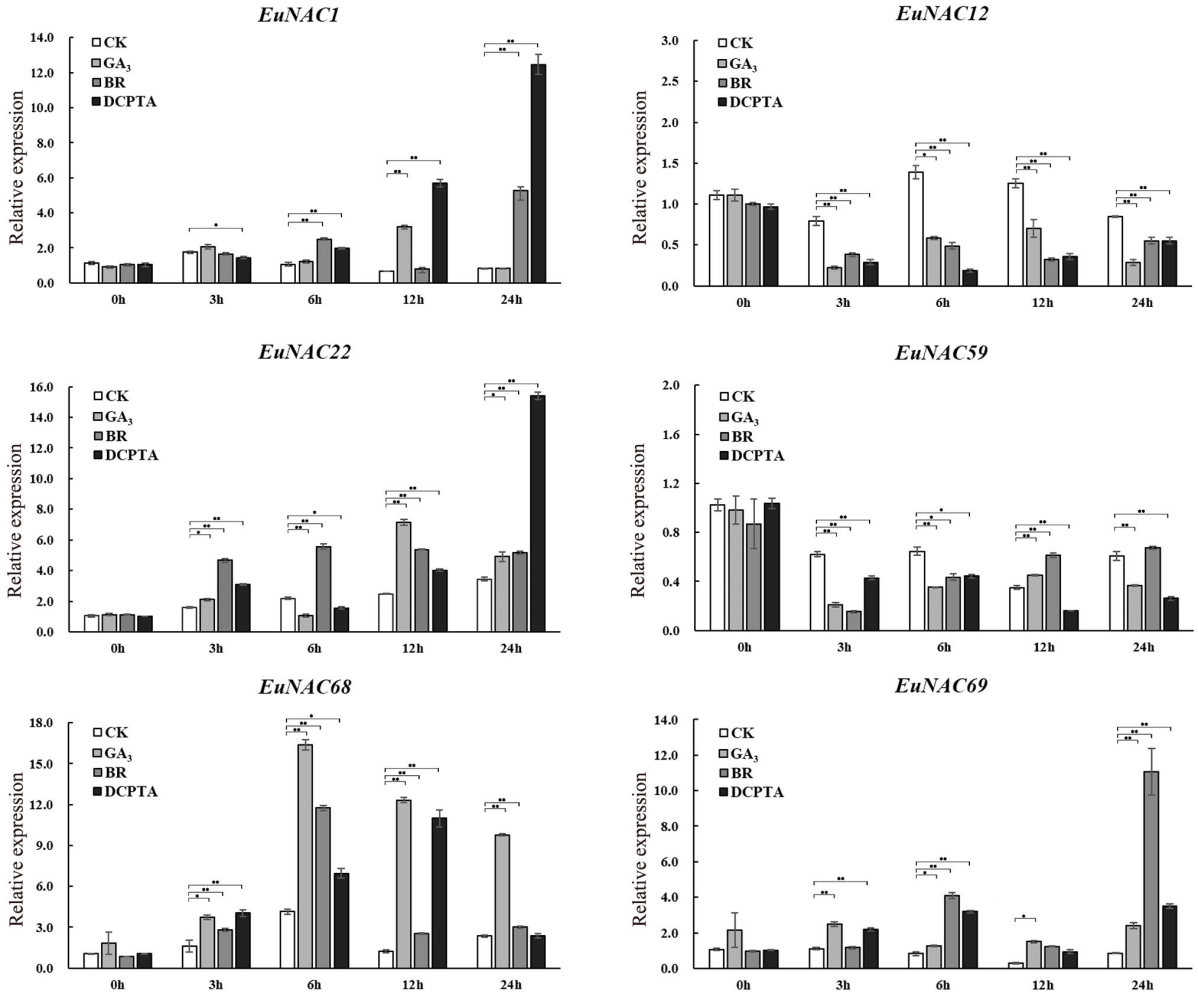
The expression patterns of *E. ulmoides* NAC genes under control condition and hormone treatments were examined by qRT-PCR. Five-month-old seedling leaves of *E. ulmoides* were sprayed with 300 mg/L gibberellin (GA3), 5 mg/L 1% brassinolide (BR), and 500 mg/L 2-(3,4-dichlorophenoxy)-triethylamine (DCPTA) until there is liquid dripping. Spray water was used as the control treatment. Error bars were obtained from three measurements. The significance analysis was carried out using Student’s t-test (* p < 0.05, ** p < 0.01).

There were significant expression changes of these genes under different treatments (**Figure 10**). Among them, the expression levels of *EuNAC22*, *EuNAC68*, and *EuNAC69* were shown to be significantly up-regulated under GA_3_ treatment at all detected time points, while the expression levels of *EuNAC12* and *EuNAC59* were observably decreased, the expression level of *EuNAC1* was only significantly increased at 12 h after GA_3_ treatment. Under BR treatment, the expression level of *EuNAC1* significantly increased 6 h and 24 h after treatment, the expression levels of *EuNAC2*2 and *EuNAC68* significantly increased at all subsequent time points after treatment (except for 3 h after treatment), and the expression level of *EuNAC69* was significantly induced. The expression level of *EuNAC59* was not different from the control at 24 h after treatment, and *EuNAC12* and *EuNAC59* were significantly inhibited at other time points. Under DCPTA treatment, the expression of *EuNAC1* and *EuNAC22* were highly induced, and *EuNAC68* was significantly increased before 24 h of DCPTA treatment. Except for 12 h after treatment, the expression level of *EuNAC69* was higher than that of the control after DCPTA treatment.

*EuNAC1* and *EuNAC22* had the highest expression levels under the DCPTA treatment, *EuNAC68* had the highest expression levels under the GA_3_ treatment, and *EuNAC69* had the highest expression levels under the BR treatment. The expression of *EuNAC68* quickly reached a maximum at 6 h after GA_3_ and BR treatments but reached a maximum at 12 h after the DCPTA treatment. The expression of *EuNAC1* and *EuNAC22* was significantly up-regulated at 24 h after DCPTA treatment. In addition, *EuNAC1* and *EuNAC69* showed relative expression peaks at 24 h after BR treatment. These results suggest that the *EuNAC* genes respond to hormonal treatment and that multiple *EuNAC* genes may play important roles in the different stages of the hormone response.

## Discussion

The *NAC* family is one of the largest plant transcription factor families. The *NAC* transcription factors play a critical role in plant growth and development, secondary metabolite synthesis, biotic and abiotic stresses, and hormone signaling pathways (Wang et al., 2014a; Wang et al., 2014b; Liu et al., 2016; Chen et al., 2018). Comprehensive investigations of the *NAC* family genes have been carried out in various plant species, such as Arabidopsis, rice, maize, and soybean. However, a systematic and comprehensive analysis of the *E. ulmoides NAC* family had not yet been carried out. In this study, we identified and comprehensively analyzed the genes of the *NAC* family of *E. ulmoides*.

The number of members of the *NAC* transcription factor gene family in different species varies significantly. In this study, we used the *E. ulmoides* genome to identify 71 *EuNAC* genes; the number of *NAC* genes was similar to *C. canephora* (63) (Dong et al., 2019) and *V. vinifera* (74) (Wang et al., 2013), less than 105 members were identified in *A. thaliana* (Ooka et al., 2003), 163 members were identified in *P. trichocarpa* (Hu et al., 2010), 189 members were identified in *E. grandis* (Hussey et al., 2015). In this study, a total of 71 *NAC* genes were identified based on the *E. ulmoides* genome (size of 1.02 G) (Li et al., 2020), while the genome size of Arabidopsis is 125 Mb with 105 *NAC* members, and the genome size of rice is 466 Mb with 151 *NAC* members (Ooka et al., 2003). It was indicated that the genome size and the number of *NAC* family members are not always related. We suspected that some *EuNAC* genes were lost during evolution, and similar evolutionary loss events occurred in the WRKY and GLK transcription factor family (Liu et al., 2021; Liu et al., 2022). The diversity in the number of *NAC* gene family members in different species may be influenced genome duplication events, such as whole-genome duplication, segmental duplication, or tandem duplication (Zhang, 2003; Chang and Duda, 2012). In the study, three pairs of genes of tandem duplication and 12 segmental duplication were identified among the *EuNAC* genes. Therefore, gene duplication, especially segmental duplication, may provide the primary driving force of expansion of *EuNAC*. This result indicated that, although some *EuNAC* genes were lost during evolution, the sufficient genetic diversity has been retained in *E. ulmoides*.

The number of *NAC* gene family members identified in different plants is varied. We constructed a phylogenetic tree for *E. ulmoides* and *A. thaliana* and divided the 71 *EuNAC* genes into 17 subgroups according to the classification of *NAC* proteins in *A. thaliana*, including one *E. ulmoides*-specific subgroup. But the result is inconsistent with other species, such as tomato (12 subgroups) (Kou et al., 2014), *Dactylis glomerata* (14 subgroups) (Yang et al., 2021), *Dimocarpus longan* (12 subgroups) (Munir et al., 2020), and *Pyrus bretschneideri* (38 subgroups) (Gong et al., 2019). This suggests that although the *NAC* gene family has similar origins, evolution differs between species. In addition, only five and 10 with the collinear relationship were found in *O. sativa* and *S. bicolor*. However, we identified 58, 26, 46, and 87 orthologous pairs in dicotyledonous plants *A. thaliana*, *V. vinifera*, *C. canephora*, and *H. brasiliensis*, respectively. These results show that the *EuNAC* genes had higher similarity and a closer evolutionary relationship with dicotyledons.

The various conserved motifs may be related to particular functions (Ooka et al., 2003; Jensen et al., 2010; Chen et al., 2011). The predicted 10 motifs are located at the N-terminal, comprising A–E subdomains (**Figures 2B**, **3**). Motifs 1–6 were the most conserved, among which motifs 2 and 6 were considered as subdomain C, and motif 1 was considered as subdomain D, which may be responsible for DNA binding (Olsen et al., 2005). Moreover, subdomain A was represented by motif 3, which may be involved in dimerization (Ernst et al., 2004; Khedia et al., 2018). Motif 4 is considered subdomain B, and motif 5 is considered subdomain E, which is thought to be responsible for the functional diversity of the NAC proteins (Ooka et al., 2003). Motifs 8 and 9 were only found in *ONAC003* (**Figure 2B**), which was consistent with the results in the *NAC* family members of kiwifruit (Jia et al., 2021) and orchardgrass (Yang et al., 2021), indicating that it is a typical component of these subgroups and may play an important role in stress responses and secondary cell wall formation (Hussey et al., 2011; Fang et al., 2015; Zhong et al., 2021b). These results confirm the classification of the *EuNAC* gene family and facilitate further study on the function of *EuNAC* genes. Most of the *EuNAC* had two introns and three exons (**Figure 2D**) and genes within the same phylogenetic clade (**Figure 2A**) have a similar number of exons. These results are consistent with poplar (Hu et al., 2010) and cucumber (Liu X. et al., 2018) *NAC* genes suggesting that the genetic makeup of *NAC* genes are similar with the previously reported species.

The same subgroup may have similar biological activities and functions (Li et al., 2021; Xu et al., 2021). The 71 *EuNAC* genes were divided into 17 groups, and the gene structure and motif arrangement of the same group of genes were similar (**Figures 1**, **2**). It was possible to predict the functions of *E. ulmoides NAC* genes based on the functions of their Arabidopsis orthologues, which could also be potentially utilized for further functional studies (Lim et al., 2012). For example, *NAP* is related to leaf senescence (Guo and Gan, 2006), floral morphogenesis (Sablowski and Meyerowitz, 1998), and salt stress (Seok et al., 2017). *Eucommia ulmoides* had seven genes (*EuNAC5*, *EuNAC9*, *EuNAC15*, *EuNAC17*, *EuNAC18*, *EuNAC42* and *EuNAC62*) in this group, which may also have these features. *ANAC019* (*AT1G52890*), *ANAC055* (*AT3G15500*), and *ANAC072* (*AT4G27410*) belong to the *AtNAC3* subgroup, their expression is induced by drought, high salinity, and ABA (Tran et al., 2004). Therefore, we speculate that *EuNAC*10 in the same subgroup is a drought and high salt responsive gene, which regulates the survival of *E. ulmoides* under adverse growth conditions.

In addition, transcription factors usually play a key role in controlling the expression of tissue-specific genes (Choi et al., 2006; Lim et al., 2012; Siew et al., 2016). This study provides useful clues for understanding gene function concerning specific processes. For instance, Group D had 10 genes that exhibited a higher expression level in the xylem than in other tissues. It is worth noting that these 10 genes have high homology with secondary wall-associated NAC (*SWN*) transcription factors (**Supplementary Table S2**), including *SND*, *NST*, and *VND*, which play critical and dominant roles in secondary cell wall biosynthesis (Hussey et al., 2011; Zhou et al., 2014; Zhang et al., 2020). These results demonstrate that the *EuNAC* genes in Group D might affect the lignin synthesis of *E. ulmoides*.

The cis-acting elements which were the binding regions of transcription factors play an important role in regulating gene expression (Liu et al., 2016; Kaur et al., 2017). There are four cis-acting elements in the *EuNAC* gene promoter: light-responsive elements, stress-responsive elements, hormone-responsive elements, and plant growth and development-related elements. Light-responsive elements are ubiquitous cis-acting elements in the *EuNAC* promoter, suggesting that light of different colors and intensities may regulate the expression of *EuNAC* genes through different pathways. Previous studies have shown that *MYB26* was the upstream regulator of secondary wall-associated NAC (*SWN*) genes (Yang et al., 2007). Among the promoters of 71 *EuNAC* genes, 69 *EuNAC* genes contained MYB elements and 66 *EuNAC* genes contained MYC elements, indicating that MYB and MYC may be important upstream regulators. The ubiquitous MYB and MYC elements in *EuNAC* gene promoters indicate that many other MYBs and MYCs may regulate their expression by combining with *EuNAC* gene promoters. The regulation of *NAC* gene expression by plant hormones has been reported for many plants (Ohnishi et al., 2005; Ji et al., 2014; Kou et al., 2014; Nieuwenhuizen et al., 2015). These are inseparable from the fact that the promoter of the NAC gene has corresponding cis-acting elements. For example, a gibberellic acid-responsive element in the *PeNAC1* promoter was required for response to gibberellic acid which influenced the salt-stress signaling pathway (Wang et al., 2016). In this study, 920 (34.43%) elements were involved in the response to plant hormones in the promoters of *EuNAC* genes (**Figure 7**); *EuNAC* genes were also associated with different hormone response classes, including 69 genes in MeJA, 58 genes in ABA, 53 genes in ethylene, and 34 genes in GA responsive cis-elements. This implied that the expression of *EuNAC* genes might be induced by ABA, MeJA, ethylene and GA_3_, *EuNAC* genes may play a central role in plant specific hormone signaling responses. Furthermore, the cis-acting elements and qRT-PCR analysis indicated that *EuNAC1*, *EuNAC12*, *EuNAC22*, *EuNAC59*, *EuNAC68* and *EuNAC69* responded to GA_3_ treatment might be dominated by cis-acting elements in the promoter region.

Some studies have reported that *NAC* transcription factors play important regulatory roles in plant natural rubber biosynthesis. For example, previous studies have found that *HbNAC1* regulates natural rubber synthesis by interacting with natural rubber synthesis-related genes in *H. brasiliensis* (Cao et al., 2017). Eu-rubber is an important natural rubber, similar to *H. brasiliensis* natural rubber, which is composed of trans-polyisoprene and cis-polyisoprene, respectively. To explore the effect of *EuNAC* genes on Eu-rubber biosynthesis, a co-expression regulatory network including Eu-rubber biosynthesis genes and *EuNAC* was constructed. We found that the positive regulation between *EuNAC* and Eu-rubber biosynthesis genes is dominant. *EuNAC22*, *EuNAC1*, *EuNAC68*, *EuNAC69*, *EuNAC12*, and *EuNAC59* have the highest degree of connectivity in the co-expression network, and *EuNAC22*, *EuNAC1*, *EuNAC68*, and *EuNAC69* had the same expression trend as the Eu-rubber synthetic structural genes of *E. ulmoides*, but *EuNAC12* and *EuNAC59* had opposite expression trends. Interestingly, the transcript levels of *EuNAC69*, *EuNAC68*, *EuNAC1*, and *EuNAC22* were consistent with the variation in Eu-rubber content (Wuyun et al., 2018; Li et al., 2020), that is, high expression in leaves and peel, but less expression in xylem and seeds. However, the expression level of *EuNAC12* and *EuNAC59* in the xylem and seeds was significantly higher than in leaves and peels. In summary, co-expression analysis was consistent with the results of expression profiles in different tissues, suggesting that *EuNAC22*, *EuNAC1*, *EuNAC68*, *EuNAC69*, *EuNAC12*, and *EuNAC59* may play important roles in Eu-rubber biosynthesis.

It has been reported that the application of exogenous hormones, such as DCPTA, GA_3_ and BR, increases Eu-rubber concentration in *E. ulmoides* leaves (Liu H. et al., 2018). To explore the role of *EuNAC* genes in hormone response and Eu-rubber biosynthesis, we focused on the expression levels of *EuNAC1*, *EuNAC12*, *EuNAC22*, *EuNAC59*, *EuNAC68*, and *EuNAC69* under exogenous GA_3_, BR, and DCPTA treatment. Six *EuNAC* genes responded to hormone treatments, but each gene had different expression patterns under different treatments. *EuNAC68* had higher expression levels at 6 h with GA_3_ and 6 h with BR treatment, which then began to decrease, thus indicating that they may play different roles in the early and late stages of the stress response. *EuNAC1*, *EuNAC22* and *EuNAC69* reached a maximum at 24 h DCPTA treatment, whereas *EuNAC68* had higher expression levels at 12 h, which shows that the *EuNAC* genes have different sensitivities to hormone treatment. The expression levels of *EuNAC1*, *EuNAC22*, EuA*NC68*, and *EuNAC69* were observably increased by GA_3_, BR, and DCPTA treatments. However, *EuNAC12* and *EuNAC59* expression levels were significantly decreased under all hormone treatments (**Figure 9**). These results indicate that the expression of *EuNAC* genes was induced and inhibited to different degrees under different hormone treatment conditions, the phenotypes induced by hormone treatment were consistent with the expression trends of *EuNAC1*, *EuNAC22*, *EuNAC68*, and *EuNAC69* but opposite to *EuNAC12* and *EuNAC59*. In conclusion, these findings supported the view that *EuNAC* may positively or negatively affect Eu-rubber biosynthesis.

Furthermore, many *NAC* transcription factors have been reported to activate the transcription of target genes by binding to the NACRS core cis-acting element (CACG or CATGT) at the promoter region (Fujita et al., 2004; Xu et al., 2013). *ANAC019*, *ANAC055* and *ANAC072* can bind to the core DNA binding element CACG in the promoter region of the drought-induced gene *ERD1* (Tran et al., 2004). Moreover, *CpNAC1* specifically binds to the NACBS element in the *CpPDS2/4* promoter to modulate carotenoid biosynthesis (Fu et al., 2016). *HbNAC1* was also found to bind to the cis-acting element CACG in the promoter region of *SRPP* in *H. brasiliensis* to regulate natural rubber synthesis (Cao et al., 2017). By analysing the promoters of the genes involved in the biosynthesis of Eu-rubber, it was observed that many genes contain multiple NACRS cis-acting elements, including the CACG element and CATGT element. Exceptions to this include *GGPS6* (evm.model.Chr8.585) and *FPS5* (Novel08257), which have only two CACG elements, and the *NgBR-like* protein (evm.model.Chr17.181), which has a single CACG element (**Supplementary Table S8**). It was deduced that *EuNACs* might regulate the synthesis of Eu-rubber by combining with the promoter of a gene involved in Eu-rubber biosynthesis. These results provide new insight and can be useful for further verification of the *EuNAC* gene functions in Eu-rubber biosynthesis.

## Conclusion

In this study, 71 *EuNAC* genes were identifed from the *E. ulmoides* genome, which were unevenly distributed on 16 chromosomes. Based on the phylogenetic tree, all the *EuNAC* genes were divided into 17 subfamilies. A comprehensive analysis of gene structure, motif composition, chromosomal distribution, gene duplication, phylogenetic, and cis-acting elements in promoters and homologous relationships were investigated. In addition, the expression of *EuNAC* genes in different tissues, co-expression network analysis and responds to various phytohormones implied that six *EuNAC* genes may participate in the biosynthesis of Eu-rubber. In the future, more comprehensive and in-depth studies on the functional properties of the *EuNAC* genes will be required. The results of this study provides valuable information for further study on the molecular mechanism of *EuNAC* genes in the biosynthesis of Eu-rubber.

## Data availability statement

The datasets presented in this study can be found in online repositories. The names of the repository/repositories and accession number(s) can be found in the article.

## Author contributions

YL and XK conceived and designed the research, SZ performed the experiments. TX, YR and ZL analyzed the data, LS provided plant materials. SZ wrote the manuscript, YL revised the manuscript. All authors contributed to the article and approved the submitted version.
