# Supplementary figures and images for: The NAC transcription factor family in Eucommia ulmoides: Genome-wide identification, characterization, and network analysis in relation to the rubber biosynthetic genes
Source: Front Plant Sci. 2023 Apr 3;14:1030298. doi: 10.3389/fpls.2023.1030298 (PMC10106570; doi:10.3389/fpls.2023.1030298)

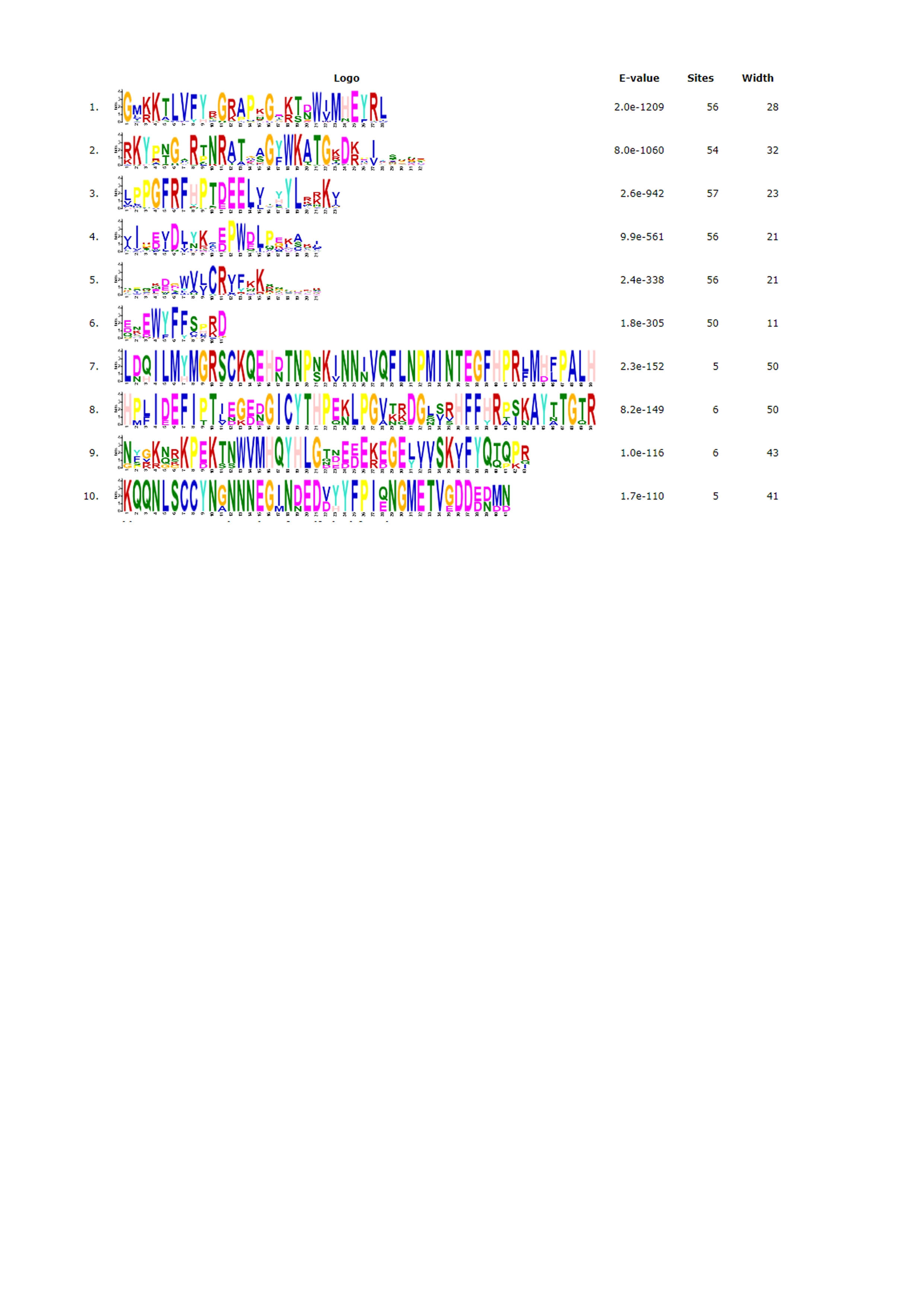


**Supplementary Figure S1. The details of the 10 putative motifs.**

Supplement: Supplementary file 1 [file DataSheet_1.zip › Figure 1.docx]
